# Gene tree correction for reconciliation and species tree inference

**DOI:** 10.1186/1748-7188-7-31

**Published:** 2012-11-20

**Authors:** Krister M Swenson, Andrea Doroftei, Nadia El-Mabrouk

**Affiliations:** 1 Département d’Informatique et de Recherche Opérationnelle, Université de Montréal, CP 6128 succ Centre-Ville, Montréal, H3C 3J7, Québec, Canada; 2 Departement of Computer Science, McGill University, 3480 University Street, Montréal, H3A 2A7, Québec, Canada

**Keywords:** Gene tree, Species tree, Reconciliation, Error correction, Maximum agreement subtree (MAST)

## Abstract

**Background:**

Reconciliation is the commonly used method for inferring the evolutionary scenario for a gene family. It consists in “embedding” inferred gene trees into a known species tree, revealing the evolution of the gene family by duplications and losses. When a species tree is not known, a natural algorithmic problem is to infer a species tree from a set of gene trees, such that the corresponding reconciliation minimizes the number of duplications and/or losses. The main drawback of reconciliation is that the inferred evolutionary scenario is strongly dependent on the considered gene trees, as few misplaced leaves may lead to a completely different history, with significantly more duplications and losses.

**Results:**

In this paper, we take advantage of certain gene trees’ properties in order to preprocess them for reconciliation or species tree inference. We flag certain duplication vertices of a gene tree, the “non-apparent duplication” (NAD) vertices, as resulting from the misplacement of leaves. In the case of species tree inference, we develop a polynomial-time heuristic for removing the minimum number of species leading to a set of gene trees that exhibit no NAD vertices with respect to at least one species tree. In the case of reconciliation, we consider the optimization problem of removing the minimum number of leaves or species leading to a tree without any NAD vertex. We develop a polynomial-time algorithm that is exact for two special classes of gene trees, and show a good performance on simulated data sets in the general case.

## Background

Almost all genomes which have been studied contain genes that are present in two or more copies. Duplicated genes account for about 15% of the protein coding genes in the human genome, for example [1]. In practise, homologous gene copies (*e.g.* copies in one genome or amongst different genomes that are descended from the same ancestral gene) are identified through sequence similarity; using a BLAST-like method, all gene copies with a similarity score above a certain threshold would be grouped into the same *gene family*. Using a classical phylogenetic method, a *gene tree*, representing the evolution of the gene family by local mutations, can then be constructed based on the similarity scores. However, macroevolutionary events (duplications, losses, horizontal gene transfer) affecting the number and distribution of genes among genomes [2], are not explicitly reflected by this gene tree. Having a clear picture of the speciation, duplication and loss mechanisms that have shaped a gene family is however crucial to the study of gene function. Indeed, following a duplication, the most common occurrence is for only one of the two gene copies to maintain the parental function, while the other becomes non-functional (pseudogenization) or acquires a new function (neofunctionalization) [3].

The most commonly used methods to infer evolutionary scenarios for gene families are based on the *reconciliation* approach that compares the species tree *S* (describing the relationships among taxa) to the gene tree *T*. Assuming no sequencing errors and a “correct” gene tree, the incongruence between the two trees can be seen as a footprint of the evolution of the gene family through processes other than speciation, such as duplication and loss. The concept of reconciling a gene tree to a species tree under the duplication-loss model was pioneered by Goodman [4] and then widely accepted, utilized and also generalized to models of other processes such as horizontal gene transfer [5-7]. Several definitions of reconciliation exist in the literature, one of them expressed in term of “tree extension” [8]. More precisely, a *reconciliationR* between *T* and *S* is an extension of *T* (obtained by grafting new subtrees onto existing edges of *T*) *consistent* with the species tree (*i.e.* reflecting the same phylogeny). A duplication and loss history for the gene family is then directly deduced from *R*. As many reconciliations exist, a natural approach is to select the one that optimizes a given criterion. Natural combinatorial criteria are the number of duplications (duplication cost), losses (loss cost) or both combined (mutation cost) [9,10]. The so called Lowest Common Ancestor (LCA) mapping between a gene tree and a species tree, formulated in [11,12] and widely used [2,10,12-16], defines a reconciliation that minimizes both the duplication and mutation costs. It has been shown in [8] that minimizing duplications follows from minimizing losses (*i.e.* a reconciliation minimizing losses also minimizes duplications, but the converse is false). When no preliminary knowledge on the species tree is given, a natural problem, known as the *species tree inference problem*, is to infer, from a set of gene trees, a species tree leading to a parsimonious evolution scenario, for a given cost. Similar to the case of a known species tree, methods have been developed for the duplication and mutation costs [9,10,17]. For both criteria, the problem of inferring an optimal species tree given a set of gene trees is NP-hard [10].

The main criticism of reconciliation is that the inferred duplication and loss history for a gene family is strongly dependent on the gene tree considered for this family. Indeed, a few misplaced leaves in the gene tree can lead to a completely different history, possibly with significantly more duplications and losses [18]. Reconciliation can therefore inspire confidence only in the case of a well-supported gene tree. Typically bootstrapping values are used as a measure of confidence in each edge of a phylogeny. How should the weak edges of a gene tree be handled? One reasonable answer is to transform the binary gene tree into an unresolved gene tree by removing each weak edge and collapsing its two incident vertices into one. Extensions of the duplication-loss model to non-binary gene trees have been considered [19,20]. Another strategy adopted in [9] is to explore the space of gene trees obtained from the original gene tree *T* by performing Nearest Neighbor Interchanges (NNIs) around weakly-supported edges. The problem is then to select, from this space, the tree giving rise to the minimum reconciliation cost.

In this paper, we explore a different strategy for “correcting” or preprocessing a gene tree *T*, prior to reconciliation or species tree inference. Criteria for identifying potentially misplaced leaves were given in [8]. The duplication vertices of *T* with respect to a species tree *S* can be subdivided into apparent and non-apparent duplication (NAD) vertices, where the latter class has been flagged as potentially resulting from the misplacement of leaves in the gene tree. The reason is that each one of the NAD vertices reflects a phylogenetic contradiction with the species tree that is not due to the presence of duplicated gene copies. In the case of an unknown species tree, we showed in [8] that deciding whether *T* can be explained using only apparent duplications (we say that *T* is an MD-tree) can be done in polynomial time, as well as inferring an appropriate species tree. Here, we present algorithmic results for removing, for a given gene tree (or a forest of gene trees), the minimum number of leaves or leaf-labels (species) leading to a tree without any NAD vertex, in both cases of a known or an unknown species tree. The minimum leaf removal problem in case of a known species tree has been recently proved to be APX-hard [21].

In the next section, we begin by formally introducing our concepts. We then motivate and state our problems in Section “Motivations and problem statements”. Section “Minimum species removal inference and recon-ciliation” gives a greedy heuristic for the minimum species removal problem in the case of an unknown species tree, and shows that any algorithm for this case can be applied to the case where the species tree is known. Section “Algo-rithms for the minimum removal reconciliation problems” is dedicated to the algorithmic developments in the case of a known species tree. We first describe two special classes of gene trees which lead to an exact polynomial-time algorithm. We then present a heuristic algorithm for the general case. In Section “Empirical results”, we test the optimality of our algorithm for the minimum leaf-removal problem in the case of a known species tree, and the ability of the presented approach to identify misplaced genes. This paper is an extended version of [22].

## Definitions

### Trees

**Figure 1:**
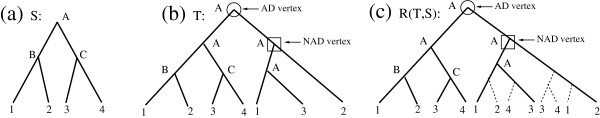
**(a) A species tree *S *for *G*={1,2,3,4}.** The three internal vertices of *S* are named *A*, *B* and *C*; **(b)** A gene tree *T*. A leaf label *x* indicates a gene copy in genome *x*. Internal vertices are labelled according to the LCA mapping between *T* and *S*. Flagged vertices are duplication vertices of *T* with respect to *S* (Section “LCA Mapping”); **(c)** A reconciliation *R*(*T*,*S*) of *T* and *S*. Dotted lines represent subtree insertions. The correspondence between vertices of *R*(*T*,*S*) and *S* is indicated by vertex labels. Flagged vertices are duplication vertices. All other internal vertices of *R*(*T*,*S*) are speciation vertices. This reconciliation reflects a history of the gene family with two gene duplications preceding the first speciation event, and 4 losses.

In this paper, we only consider rooted trees. Let *G*={1,2,⋯,*g*} be a set of integers representing *g* different species (genomes). A ***species tree*** on *G* is a rooted binary tree with exactly *g* leaves, where each *i* ∈ *G*is the label of a single leaf (Figure 1a). A ***gene tree*** on *G* is a rooted binary tree where each leaf is labelled by an integer from *G*, with possibly repeated leaves (Figure 1b). A gene tree represents a gene family, where each leaf labelled *i* represents a gene copy located on genome *i*. In the case of a species tree or a ***uniquely leaf-labelled gene tree*** (*i.e.* no leaf-label occurring more than once) we will make no distinction between a leaf and its label.

Given a tree *U*, the ***size of ****U*, denoted by |*U*|, is the number of leaves of *U*, and the ***genome set of ****U*, denoted by L(U), is the subset of *G* defined by the labels of the leaves of *U*. Given a vertex *x* of *U*, *U*_*x*_ is the subtree of *U* rooted at *x*. The genome set of *U*_*x*_ is denoted by L(x) (for example, in the tree of Figure 1a, L(B)={1,2}). If *x* is not a leaf, we denote by *x*_*l*_ and *x*_*r*_ the two children of *x*. Finally, if *x* is not the root, any vertex *y*≠*x*on a path from *x* to the root is an ***ancestor*** of *x*.

Given a tree *U* on a genome set *G*, a ***leaf removal*** consists of removing a given leaf from *U*, along with its parental node *x*, and if *x* is not the root joining the parent of *x* and the remaining child by a new edge. A tree $U^{\prime }$ obtained from *U* through a sequence of leaf removals is said to be ***included in****U*. The tree *U****restricted to a subset***$G^{\prime }$ of *G* is the tree $U^{\prime }$ obtained from *U* through a sequence of leaf removals that removes all the leaves with labels in $G\setminus G^{\prime }$.

Finally, a subtree *U*_*x*_of *U*, for a given vertex *x*, is said to be a ***maximum subtree*** of *U* verifying a given property P if and only if *U*_*x*_ verifies property P and, for any vertex *y* that is an ancestor of *x*, *U*_*y*_does not verify property P.

### Reconciliation

Applying a classical phylogenetic method to the gene sequences of a given gene family leads to a gene tree *T* that is different from the species tree *S*, mainly due to the presence of multiple gene copies in *T*, and that may reflect a divergence history different from *S*. The reconciliation approach consists in “embedding” the gene tree into the species tree, revealing the evolution of the gene family by duplications and losses.

There are several definitions of reconciliation between a gene tree and a species tree [2,10-15]. Here we define reconciliation in terms of subtree insertions, following the notation used in [14,23]. We begin with some definitions:

A ***subtree insertion*** in a tree *T* grafts a new subtree onto an existing edge of *T*. Formally, inserting a subtree onto an edge linking two nodes *x* and *y* (*y* is a child of *x*) consists in creating a new node *z* with parent *x* and two children being *y* and the root of the inserted subtree.

A tree $T^{\prime }$ is said to be an ***extension*** of *T* if it can be obtained from *T* by subtree insertions on the edges of *T*.

The gene tree *T* is said to be ***DS-consistent with****S* (DS standing for Duplication/Speciation) if *T* reflects a history with no loss, *i.e.* if for every vertex *t* of *T* such that |L(t)|≥2, there exists a vertex *s* of *S* such that L(t)=L(s) and one of the two following conditions holds: 

either $L(t_{r})=L(t_{\ell })$ (indicating a Duplication),

or $L(t_{r})=L(s_{r})$ and $L(t_{\ell })=L(s_{\ell })$ (indicating a Speciation).

#### Definition 1

A **reconciliation** between a gene tree *T* and a species tree *S* on *G* is an extension *R*(*T*,*S*) of *T* that is DS-consistent with *S*.

For example, the tree of Figure 1c is a reconciliation between the gene tree *T* of Figure 1b and the species tree of Figure 1a. Such a reconciliation between *T* and *S* implies an unambiguous evolution scenario for the gene family, where a vertex of *R*(*T*,*S*) that satisfies property (D) represents a duplication (duplication vertex), a vertex that satisfies property (S) represents a speciation (speciation vertex), and an inserted subtree represents a gene loss. The number of duplication vertices of *R*(*T*,*S*) is called the ***duplication cost*** of *R*(*T*,*S*).

The notion of reconciliation can naturally be extended to the case of a set, or *forest*, of gene trees $F=\{T_{1},\ldots ,T_{f}\}$: a reconciliation between  F and *S* is a set $R(F,S)=\{R_{1}(T_{1},S),\ldots ,R_{f}(T_{f},S)\}$ of reconciliations, respectively for *T*_1_,…,*T*_*f*_, such that each *R*_*i*_(*T*_*i*_,*S*) is DS-consistent with *S*.

### LCA Mapping

The LCA mapping between a gene tree *T* and a species tree *S*, denoted by *M*, maps every vertex *t* of *T* to the Lowest Common Ancestor (LCA) of L(t) in *S*. A vertex *t* of *T* is called a ***duplication vertex*** of *T* with respect to *S* if and only if *M*(*t*_*ℓ*_)=*M*(*t*) and/or *M*(*t*_*r*_)=*M*(*t*) (see Figure 1b). We denote by **d(T,S)**the number of duplication vertices of *T* with respect to *S*. Any vertex of *T* that is not a duplication vertex is a ***speciation vertex*** with respect to *S*.

The LCA mapping induces a reconciliation *M*(*TS*) between *T* and *S*, where an internal vertex *t* of *T* leads to a duplication vertex in *M*(*TS*) if and only if *t* is a duplication vertex of *T* with respect to *S*. In other words, the duplication cost of *M*(*TS*) is *d*(*TS*) (see for example [10,13,15] for more details on the construction of a reconciliation based on the LCA mapping). Moreover, *M*(*TS*) is a reconciliation that minimizes the duplication, loss, and mutation costs [8,14].

### Duplication vertices and MD-trees

Let *T* be a gene tree. As noticed in [8], any vertex *t* of *T* such that $L(t_{\ell })\cap L(t_{r})\neq \emptyset$ (*i.e.* the left and right subtrees rooted at *t* contain a gene copy in the same genome) will be a duplication vertex in any reconciliation between *T* and any species tree *S*, in particular in *M*(*TS*). Such a vertex is called an ***Apparent Duplication vertex*** (***AD vertex*** for short) of *T*. In the tree of Figure 1b, the root is an AD vertex as its left and right subtree both contain a gene copy in genome 1. Following our notation in [8], a gene tree *T* is said to be a ***Minimum Duplication Tree*** (henceforth called an ***MD-tree***) if there exists a species tree *S* such that *d*(*TS*) is exactly the number of apparent duplications present in *T*. In which case, *T* is said to be ***MD-consistent*** with *S*.

However, this is not always true, in other words, a duplication vertex of *T* with respect to a species tree *S* is not necessarily an AD vertex. We call such a duplication vertex a ***Non-Apparent Duplication vertex***, or simply a ***NAD vertex***. For example, the tree of Figure 1b contains one NAD vertex, indicated by a square, and thus *T* is not MD-consistent with *S*.

## Motivations and problem statements

Non-apparent duplication vertices point to disagreement between a gene tree *T* and a species tree *S* that are not due to the presence of repeated leaf labels (*i.e.* multiple copies in the same genome). More precisely, we say that a vertex *x* of *Tsplits* three species {*abc*} into {*ab*;*c*} if the genome set of one of its children contains *a* and *b* but not *c*, and the genome set of its other child contains *c* but neither *a* nor *b*. Then for any NAD *x* of *T*, there is a triplet of species {*abc*} that are split differently by *x* and by the LCA mapping of *x* in *S* (see proof of Theorem 5 in [8]). We will say that such a triplet *exhibits a wrong phylogeny*. For example, in Figure 1, {1,2,3} is split into {1,3;2} by the NAD vertex of *T*, and into {1,2;3} by the vertex *A* in *S*. It has therefore been suggested that NAD vertices may point at gene copies that are erroneously placed in the gene tree.

Observations made in [8] tend to support this hypothesis. In particular, using simulated data-sets based on the species tree of 12 *Drosophila* species given in [24] and a birth-and-death process, starting from a single ancestral gene, and with different gene gain/loss rates, it has been found that 95% of gene duplications lead to an AD vertex.

**Figure 2:**
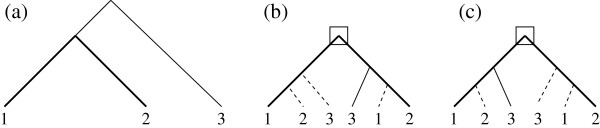
**Let *S*=((1,2),3) (the tree in (a)) be the phylogenetic tree for the three species {1,2,3}.** Let *T*=(1,2) be a gene tree. **(a)**, **(b)** and **(c)** are the three possibilities for *T* after a random insertion of a leaf labelled 3. **(a)** is the only case leading to a tree without any NAD vertex. It reflects a history of the three gene copies without any duplication or loss; **(b)** and **(c)** each contains a NAD vertex, and can be explained by a duplication-loss history of minimum mutation cost of 4: 1 duplication and 3 losses.

Notice however that a misplaced gene in a gene tree *T* does not necessarily lead to a NAD vertex. In other words, NAD vertices can only point to a subset of misplaced leaves. However, in the context of reconciliation, the damage caused by a misplaced leaf leading to a NAD vertex is to significantly increases the real mutation-cost of the tree, as shown in Figure 2.

Following the later observations, we exploit the properties of NAD vertices for gene tree correction. For generality, we consider a forest of gene trees $F=\{T_{1},\ldots ,T_{f}\}$. If  F is not MD-consistent with a given species tree *S* (*i.e.* there is at least one tree in  F that is not MD-consistent with *S*) then an MD-consistent forest can always be obtained from  F by performing a certain number of leaf removals. Indeed, a gene tree with only two leaves is always MD-consistent with any species tree. Our first optimization problem is the following, where the size of  F is just the sum of sizes of all the trees of  F.

MINIMUM LEAF REMOVAL RECONCILIATION(MINLRR):**Input:** A genome set *G*, a forest of gene trees $F=\{T_{1},\ldots ,T_{f}\}$ on *G*, and a species tree *S* for *G*; **Output:** A forest of gene trees $F^{\mathrm{MAX}}=\{T_{1}^{\mathrm{MAX}},\ldots ,$$T_{f}^{\mathrm{MAX}}\}$ of maximum size (*i.e.* obtained from  F by a minimum number of leaf removals) which is MD-consistent with *S*, where each $T_{i}^{\mathrm{MAX}}$ is included in *T*_*i*_.

In the case of an unknown species tree, we have shown in [8] that deciding whether a forest of gene trees  F is an MD-forest (*i.e.* a set of MD-trees) can be done in polynomial time and space, as well as computing a parsimonious species tree. For a forest which is not an MD-forest, a natural generalization of the MINLRR problem is the following:

MINIMUM LEAF REMOVAL INFERENCE (MINLRI):**Input:** A genome set *G* and a forest of gene trees $F=\{T_{1},\ldots ,T_{f}\}$ on *G*; **Output:** An MD-forest $F^{\mathrm{MAX}}=\{T_{1}^{\mathrm{MAX}},\ldots ,T_{f}^{\mathrm{MAX}}\}$ of maximum size (*i.e.* obtained from  F by a minimum number of leaf removals), where each $T_{i}^{\mathrm{MAX}}$ is included in *T*_*i*_.

A more conservative strategy that can be used to reduce the risk of inferring a wrong species tree, is to remove the minimum number of species from *G* such that the forest  F restricted to the new genome set is an MD-forest. Removing the minimum number of species instead of leaves can also be considered in the case of reconciliation, a scenario that may be applicable when full confidence is not put in the species tree.

MINIMUM SPECIES REMOVAL RECONCILIATION (MINSRR):**Input:** A genome set *G*, a forest of gene trees $F=\{T_{1},\ldots ,T_{f}\}$ on *G* and a species tree *S* for *G*; **Output:** A maximum subset $G^{\prime }$ of *G* such that forest  F restricted to $G^{\prime }$ (*i.e.* the set of trees *T*_*i*_restricted to $G^{\prime }$) is MD-consistent with the species tree *S* restricted to $G^{\prime }$.

MINIMUM SPECIES REMOVAL INFERENCE(MINSRI):**Input:** A genome set *G* and a forest of gene trees $F=\{T_{1},\ldots ,T_{f}\}$ on *G*; **Output:** A maximum subset $G^{\prime }$ of *G* such that the forest  F restricted to $G^{\prime }$ is an MD-forest.

The latter two optimization problems (MINSRR and MINSRI)) are the subject of the next section. Section “Algorithms for the minimum removal reconcilia-tion problems” focuses on the two optimization problems related to reconciliation (MINLRR and MINSRR).

## Minimum species removal inference and reconciliation

By linking the species tree inference problem to a supertree problem we have been able to prove that deciding whether a gene tree *T* is an MD-tree can be done in polynomial-time [8]. We used a constructive proof based on a min-cut strategy, which has been largely considered in the context of supertrees [25-27]. In this section, we develop a greedy heuristic for MINSRI based on a minimum vertex cut strategy.

Let $F=\{T_{1},T_{2},\ldots ,T_{f}\}$ be a forest of gene trees on a genome set *G*. Define $\mathrm{leve}l_{0}(F)$ to be the set of highest (*i.e.* closest to the root) vertices of all *T*_*i*_s that are not AD-vertices. $\mathrm{leve}l_{j}(F)$ is then the set of vertices of all *T*_*i*_s that are closest non-AD descendants of the vertices for $\mathrm{leve}l_{j-1}(F)$. For a given level *j*, forest  F, and vertex $x\in \mathrm{leve}l_{j}(F)$, consider the bipartition $B(x)=(L(x_{l}),L(x_{r}))$. Then $G_{j}=(V,E)$ is the corresponding hypergraph [28] where *V*=*G*, and $L(x_{l}),L(x_{r})\in E$ for $x\in \mathrm{leve}l_{j}(F)$.

In order for  F to be an MD-forest, all the vertices of $\mathrm{leve}l_{j}(F)$, for any *j*, should represent speciation vertices with respect to some species tree *S* (as otherwise they would represent additional non-apparent duplication vertices, preventing the forest from being an MD-forest). In other words, the bipartitions *B*(*x*) for all *x* ∈ *level*_0_(*T*) should reveal a first speciation event, which is possible if and only if the graph $G_{0}$ contains at least two connected components. Indeed, in this case for any species tree *S* with a root *r* splitting *G* into two disconnected subsets, all the vertices of $\mathrm{leve}l_{0}(F)$ would be speciation vertices. Conversely, if $G_{0}$ contains a single connected component, then for any species tree *S*, at least one node of *level*_0_(*T*) would be a NAD node. The same reasoning applies to any $\mathrm{leve}l_{j}(F)$ and $G_{j}$.

On the other hand, if $G_{j}$ is connected for some $\mathrm{leve}l_{j}(F)$, there exists no species tree so that all $x\in \mathrm{leve}l_{j}(F)$ represent speciation events. In this case, some number of species must be removed to make $G_{j}$ disconnected. This corresponds exactly to a vertex cut in $G_{j}$. These observations leads to the following heuristic for the MINSRI problem.

ALGORITHMMINIMUM SPECIESREMOVAL INFERENCE(  F)

1. $G^{\prime }=G$; *j*=0; Compute $\mathrm{leve}l_{0}(F)$;

2. WHILE $\mathrm{leve}l_{j}(F)$ is not empty DO

3. Construct the hypergraph $G_{j}$;

4. IF $G_{j}$ is connected THEN

5. $G^{\prime }=G^{\prime }\setminus \text{MINIMUM-VERTEX-CUT}(G_{j})$;

6. Restrict  F to $G^{\prime }$;

7. END IF

8. *j*=*j* + 1;

9. Compute $\mathrm{leve}l_{j}(F)$;

10. END WHILE

11. RETURN ( $G^{\prime }$)

MINIMUM-VERTEX-CUT in a hypergraph can be computed using the minimum vertex cut algorithm for simple graphs: each hyperedge corresponds to a clique in the simple graph. It is easy to confirm that a set of vertices disconnects the hypergraph if and only if it disconnects the corresponding simple graph. Vertex cut on a simple graph can be implemented with 2*n*−2 calls to the standard *st* vertex cut algorithm (based on minimum *st* edge cut). By reusing computation, Hao and Orlin [29] showed how to do all 2*n*−2 calls to the *st* cut algorithm in the same time it takes to do a single call. Thus, MINIMUM-VERTEX-CUT can be solved in $O(|V||E|\mathrm{lg}(|V{|}^{2}/|E|)$ time. Since we call MINIMUM-VERTEX-CUT*O*(|*V*|) times in the worst case, Algorithm Minimum Species Removal Inference runs in $O(|V{|}^{2}|E|\mathrm{lg}(|V{|}^{2}/|E|)$ time.

In the next section we give algorithms for MINSRR and MINLRR.. We conclude this section by highlighting the relationship between MINSRR and MINSRI.

### Remark 1

MINSRR reduces to MINSRI.

This is easy to see; take the species tree *S* given by the instance of MINSRR and add it to the forest  F for the MINSRI problem. The solution to MINSRI gives a species tree that must be a subtree of *S*. Thus, any algorithm for MINSRI can be used to solve MINSRR.

## Algorithms for the minimum removal reconciliation problems

In this section, we assume that a species tree *S* is known for the genome set *G*. For simplicity, we present the algorithms in the case of a single gene tree *T*, although it is straightforward to generalize them to the case of a forest of gene trees.

Let *T* be a gene tree for a gene family on *G*. We suppose that *T* is not an MD-tree consistent with *S* (*i.e.* there is at least one duplication vertex of *T* that is a NAD vertex). We begin by describing special classes of gene trees for which exact polynomial-time algorithms have been developed for the MINLRR and MINSRR problems.

### Uniquely leaf-labelled gene trees

When the considered gene family contains at most one gene per genome, the gene tree *T* is uniquely leaf-labelled. In this case, minimizing the number of leaves, or equivalently species, that should be removed from *T* to obtain an MD-tree consistent with *S* is equivalent to finding the maximum number of genomes that lead to the same phylogeny in *T* and *S*. In other words, it is immediate to see that the MINLRR problem reduces, in this case, to the MAST problem given below.

MAXIMUM AGREEMENT SUBTREE (MAST):**Input:** A uniquely leaf-labelled gene tree *T* on *G* and a species tree *S* for *G*; **Output:** A tree *T*^*MAX*^included in *T* such that it is MD-consistent with *S* and of maximum size.

A more general definition is given in the literature, where the MAST problem is defined on a set of uniquely leaf-labelled trees as the largest tree included in each tree of the set. This definition is equivalent to ours in the case of a gene tree *T* and a species tree *S*.

The MAST problem arises naturally in biology and linguistics as a measure of consistency between two evolutionary trees over species or languages [30]. In the evolutionary study of genomes, different methods and different gene families are used to infer a phylogenetic tree for a set of species, usually yielding different trees. In such a context, one has to find a consensus of the various obtained trees. Considering the MAST problem, introduced by Finden and Gordon [31], is one way to obtain such a consensus. Amir *et al.*[32] showed that computing a MAST of three trees with unbounded degree is NP-hard. However, in the case of two binary trees, the problem is polynomial. The first polynomial-time algorithm for this problem was given by Steel and Warnow [33]. It is a dynamic programming algorithm considering the solution for all pairs of subtrees of *T* and *S*; it has a running time of *O*(*n*^2^), where *n* is the number of leaves in the trees. Later, Cole *et al.*[30] developed an O(nlgn) time algorithm, which, as far as we know, is the most efficient algorithm for solving the MAST problem on two binary trees. We use this result in the MINLRR version of our algorithms. In the case of *k* binary trees, the current fastest known algorithms run in *O*(*kn*^3^) time [34,35]. We use this result in the MINSRR version of our algorithms.

### No AD above NAD

**Figure 3:**
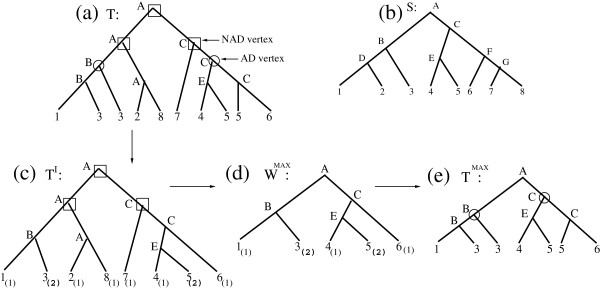
**Solving the MINIMUM LEAF REMOVAL RECONCILIATION problem for a tree satisfying CONSTRAINT C; (a) A gene tree *T *on *G*={1,2,3,4,5,6,7,8}; (b) A species tree *S *for *G*.** Internal vertices of *S* are identified with different characters. Labels of internal vertices of *T* are attributed according to the LCA mapping between *T* and *S*. *T* contains 5 duplication vertices with respect to *S*: two AD vertices (surrounded by a circle) and three NAD vertices (surrounded by a square); **(c)** The tree *T*^*I*^obtained by replacing the two subtrees of *T* rooted at each of the two AD vertices by their weighted induced trees. Leaves’ weights are given in brackets; **(d)** The weighted agreement subtree *W*^*MAX*^of *T*^*I*^and *S* of maximum value. *v*(*W*^*MAX*^)=7; **(e)** The subtree *T*^*MAX*^of *T* induced by *W*^*MAX*^. *T*^*MAX*^is an MD-tree consistent with *S*.

In this section, we consider a tree *T* containing no AD vertex above a NAD vertex (Figure 3a). More precisely, *T* satisfies Constraint C below:

CONSTRAINT C: For each NAD vertex *x* of *T*, if *y* is an ancestor of *x* that is a duplication vertex, then *y* is a NAD vertex.

We show that the MINSRR problem reduces, in this case, to the MAST problem, while the MINLRR problem reduces to a “generalization” of the MAST problem to weighted trees, where a *weighted tree* is a uniquely leaf-labelled tree with weighted leaves.

#### Definition 2

Let *U* be a tree on *G*. The *weighted tree induced by* (*U,S*) is the tree included in *S* obtained from *S* by removing all leaves that are not in L(U), with a weight attributed to each leaf *s*, representing the number of occurrences of *s* in *U* (*i.e.* the number of leaves of *U* labelled *s*).

Let *T*_1_,*T*_2_,⋯*T*_*m*_ be the maximum subtrees of *T* rooted at an AD vertex (*i.e.* subtrees of *T* rooted at the highest AD vertices). Then, the tree *T*^*I*^ obtained by replacing each *T*_*i*_, for 1 ≤ *i* ≤ *m*, by the weighted tree induced by (*T*_*i*_,*S*), is a weighted uniquely leaf-labelled tree. An example is given in Figure 3a,b and c. Let *ρ*_*s*_ be the operation of removing the weighted leaf *s* from *T*^*I*^. Then the corresponding removals in *T* consist of removing from *T* all leaves labelled *s*.

Finally, we formulate the generalization of the MAST problem to weighted trees as follows, where the value *v*(*W*) of a weighted tree *W * is the sum of its leaves’ weights.

WEIGHTED MAXIMUM AGREEMENT SUBTREE(WMAST):**Input:** A weighted tree *W * on *G* and a species tree *S* for *G*; **Output:** A weighted tree *W*^*MAX*^included in *W * such that it is MD-consistent with *S* and of maximum value.

We are now ready for the main theorem.

#### Theorem 1

Let *T* be a gene tree satisfying CONSTRAINT C. Let *W*^*MAX*^be a solution of the WMAST problem on *T*^*I*^and *S*, and *T*^*MAX*^be the subtree included in *T* obtained by removing from *T* all the leaves that are not leaves of *W*^*MAX*^. Then *T*^*MAX*^is a solution of the MINLRR problem on *T* and *S*.

In other words, solving the MINLRR problem on *T* is equivalent to solving the WMAST problem on *T*^*I*^. We show in the proof of Theorem 2 that WMAST can be solved by the traditional MAST algorithms with no change in the asymptotic running time.

A complete example of the algorithmic methodology used for solving the MINLRR problem on *T* and *S* is given in Figure 3. The algorithm will be detailed in the next section.

We now provide a proof of Theorem 1, subdivided into the two following lemmas.

#### Lemma 1

The tree *T*^*MAX*^is MD-consistent with *S*.

#### Proof

We show, by contradiction, that *T*^*MAX*^does not contain any NAD vertex. Suppose that *T*^*MAX*^contains a NAD vertex *x*. Then *x* maps to the same vertex *s* of *S* than one of its children, lets say the left child. Then there exists two leaves of $T_{x_{l}}^{\mathrm{MAX}}$, labelled *a* and *b*, and one leaf of $T_{x_{r}}^{\mathrm{MAX}}$ labelled *c* such that the triplet {*a*,*b*,*c*} exhibits a wrong phylogeny. As a non-duplication vertex in *T* cannot become a duplication vertex after leaf removals, we have only two possibilities for *x* in *T*: 

1. *x is a NAD vertex in T*. Then the genome sets of $T_{x_{l}}$ and $T_{x_{r}}$ are disjoint. Moreover, the genome set of $\left(W_{x_{l}}^{\mathrm{MAX}}\right)$ (resp. $\left(W_{x_{r}}^{\mathrm{MAX}}\right)$) is a subset of the genome set of $T_{x_{l}}$ (resp. $T_{x_{r}}$). On the other hand, as *x* is not a duplication vertex in *W*^*MAX*^, one of the three genes *a*, *b* and *c* should be absent in $W_{x}^{\mathrm{MAX}}$. And thus, {*a*,*b*,*c*} can not be a subset of the genome set of $T_{x}^{\mathrm{MAX}}$, a contradiction.

2. *x is an AD vertex in T*. Then the subtree *T*_*x*_of *T* rooted at *x* contains at least two leaves labelled with the same label *d* (different from *a*, *b* and *c*), one in $T_{x_{l}}$ and one in $T_{x_{r}}$. Moreover the leaf labelled *d* in *S* should belong to the subtree of *S* rooted at *s*, and thus to the subtree *S*_*i*_rooted at the left or right child of *s*. Such subtree *S*_*i*_contains at least one leaf labelled *a* or *b* or *c*.

On the other hand, let *y* be the parent of *x* in *T*^*I*^. As an optimal solution of the WMAST problem on *T*^*I*^removes leaves from the subtree $\left(T_{x}^{I}\right)$, such an operation should result in removing the duplication vertex *y*. In other words, *x* and *y* should map to the same vertex *s* in *S*. Moreover the result of the leaf removal from $\left(T_{x}^{I}\right)$ should result in a different LCA mapping for *x* and *y*. Indeed, removing leaves from the corresponding subtree in *T*^*I*^ does not contribute to eliminating any NAD from *T*^*I*^. It follows that *S* should exhibit the phylogeny ((*a*,*b*,*c*),*d*), which is a contradiction with the result of the last paragraph. □

#### Lemma 2

Let $T^{\prime }$ be a tree included in *T* that is MD-consistent with *S*. Then $\left|T^{\prime }|\leq |T^{\mathrm{MAX}}\right|$.

#### Proof

We will show that, for any *s* ∈ *G*, if a leaf *i* labelled *s* is removed from *T* (*i.e.i* is not a leaf in $T^{\prime }$), then all leaves of *T* labelled *s* are removed from *T*.

Suppose this is not the case. Let *y* be the vertex of *T* representing the least common ancestor of all leaves labelled *s* in *T*. Then *y* is an AD node. As a leaf *i* labelled *s* is removed from *T*, such removal should contribute to resolving a NAD vertex *x* of *T*. From CONSTRAINT C, such a vertex should be outside the subtree of *T* rooted at *y*. Moreover, it should clearly be an ancestor of *y* (otherwise removing *i* will have no effect on *x*).

As *x* is a NAD vertex, it maps to the same vertex *s* of *S* as one of its children, say the left child. Then, there exist two leaves of $T_{x_{l}}$ labelled *a* and *b*, and one leaf of $T_{x_{r}}$ labelled *c* such that the triplet {*a*,*b*,*c*} exhibits a wrong phylogeny. Moreover, as removing leaf *i* labelled *s* contributes to solving *x*, we can assume that *a*=*s*. However, from our assumption, there remains a leaf labelled *s* in $T^{\prime }$. Thus: either (1) at least one leaf labelled *b* and one leaf labelled *c* remains in $T^{\prime }$, or (2) all leaves labelled *b* or all leaves labelled *c* are removed. In case (1), the wrong phylogeny exhibited by the triplet {*a*,*b*,*c*} is still present, preventing vertex *x* from being a non-duplication vertex. In case (2), as all copies of *b* (or equivalently *c*) are removed, there is no need to remove leaf *i* labelled *s* to correct the wrong phylogeny exhibited by the triple {*a*,*b*,*c*}.

Therefore, the weighted tree $W^{\prime }$ induced by $T^{\prime }$ is obtained from *T*^*I*^through a sequence of leaf removals. Now, as *W*^*MAX*^is the solution of the WMAST problem on *T*^*I*^, then $v(W^{\mathrm{MAX}})\geq v(W^{\prime })$, and thus $\left|T^{\mathrm{MAX}}|\geq |T^{\prime }\right|$. □

Finally, the following corollary makes the link between the MINSRR problem and the MAST problem.

#### Corollary 1

Let *T* be a gene tree satisfying CONSTRAINT C. Let *W*^*MAX*^be a solution to the MAST problem on *T*^*I*^and *S* (ignoring weights), and *T*^*MAX*^be the subtree of *T* induced by *W*^*MAX*^. Then *T*^*MAX*^is a solution to the MINSRR problem on *T* and *S*.

To apply the algorithm to MINSRR with the forest $F=\{T_{1},T_{2},\ldots ,T_{k}\}$, all trees $T_{i}^{I}$ must simultaneously agree with *S*, so the *O*(*kn*^3^)MAST algorithm [34,35] must be used.

### An Algorithm for the general case

In this section, we present a general algorithm, that is exact in the case of a uniquely leaf-labelled gene tree (Section “Uniquely leaf-labelled gene trees”) or a gene tree satisfying CONSTRAINT C (Section “No AD above NAD”), and a heuristic in the general case. We first introduce preliminary definitions. For a given tree *U* (weighted or not), consider the two following properties on *U*:

Property ONLY-NAD:*U* has no AD vertices; Property ONLY-AD:*U* is rooted at an AD vertex and contains no NAD vertex.

We define the ***NAD-border*** of *U* as the set of roots of the maximum subtrees of *U* verifying Property ONLY-NAD, and the ***AD-border*** of *U* as the set of roots of the maximum subtrees of *U* verifying Property ONLY-AD.

ALGORITHM CORRECT-TREE is a recursive algorithm that takes as input a gene tree *T* and a species tree *S*, and outputs a number of leaf removals transforming *T* into a tree that is MD-consistent with *S*. It proceeds as follows: 

Stop condition - Lines 2 to 4: If *T* is MD-consistent with *S*, then no leaf removal is performed, and the algorithm terminates.

Recurrence Loop - Lines 6 to 13: Resolve all maximum subtrees of *T* verifying CONSTRAINT C as described in Section “No AD above NAD”, that is: 

1. Construct the weighted tree *T*^*I*^(Lines 6-8);

2. For each root *x* of a maximum subtree $T_{x}^{I}$ of *T*^*I*^satisfying CONSTRAINT C (Line 9), solve the WMAST problem on $T_{x}^{I}$, which leads to the weighted tree $W_{x}^{\mathrm{MAX}}$ (Line 10), compute the induced tree *T*_*x*_(Line 11) and store the number of performed leaf removals (Line 12).

### Algorithm Correct-Tree (*T*, *S*)

1. LeafRemoval=0;

2. IF *T* is a tree MD-consistent with *S* THEN

3. 

 RETURN (LeafRemoval)

4. END IF

5. *T*^*I*^=*T*;

6. FOR ALL *x* ∈ AD-border(*T*) DO

7. 

 Replace $T_{x}^{I}$ by its induced weighted tree;

8. END FOR

9. FOR ALL *x* ∈ NAD-border(*T*^*I*^) DO

10. 

$\left(W_{x}^{\mathrm{MAX}}=\text{WMAST}(T_{x}^{I})\right)$;

 Replace *T*_*x*_by the subtree induced by $\left(W_{x}^{\mathrm{MAX}}\right)$;

 LeafRemoval = LeafRemoval + $\left(\left(v(T_{x}^{I})-v(W_{x}^{\mathrm{MAX}})\right)\right)$;

11. END FOR

12. RETURN (LeafRemoval+CORRECT-TREE(*T*, *S*))

If *T* is a uniquely leaf-labelled tree then *T*^*I*^=*T*, NAD-border(*T*^*I*^) is reduced to the root of the tree, and thus loop 9–13 is just executed once. Moreover, as *T*^*I*^ is unweighted (all labels are equal to 1), WMAST is reduced to MAST. The whole algorithm thus reduces to one resolution of the MAST problem.

If *T* satisfies CONSTRAINT C, then NAD-border(*T*^*I*^) is also reduced to the root of the whole tree, and thus loop 9–13 is just executed once. In this case, the methodology is the one following Theorem 1, and illustrated in Example 3.

In the general case, NAD-border(*T*^*I*^) is not restricted to a single vertex, and loop 9–13 can be executed many times. Moreover, at the end of loop 9–13, the resulting tree is not guaranteed to be MD-consistent with *S*, as NAD vertices higher than those in NAD-border(*T*^*I*^) may exist. Algorithm Correct-Tree may therefore be applied many times.

#### Theorem 2

Algorithm Correct-tree has time-complexity $O(n^{2}\mathrm{lg}n)$, where *n* is the size of *T*.

#### Proof

Let *n* be the size of *T*. Loop 2–4 requires the LCA mapping between *T* and *S*, and the identification of AD and NAD vertices. As the LCA mapping can be computed in linear time [8,36], testing whether a tree *T* is MD-consistent with *S* can be tested in time *O*(*n*). Clearly, Loop 6–8 can be executed in time *O*(*n*). As for Loop 9–13, it has the time complexity *O*(*C*) of WMAST. Therefore, the complexity for one execution of the recursive ALGORITHM CORRECT-TREE is *O*(*C*). As in the worst case the algorithm can be executed *Ω*(*n*) times, the total worst case running time is *O*(*nC*).

Let us consider the complexity of WMAST. The *O*(*n*^2^) algorithm of Steel and Warnow [33] naturally generalizes to the case of weighted trees, and leads to the same complexity, *O*(*n*^2^). However, we show in the rest of this proof that an instance of WMAST can be transformed into an instance of MAST in linear-time, which allows us to consider *C* as being the best complexity found for MAST, namely the O(nlog(n)) running-time of the algorithm given in [30].

Let *G* be a genome set, *W * be a weighted tree on *G* and *S* be a species tree for *G*. Then consider the *expanded genome setG*_*exp*_obtained from *G* by replacing each genome *g* with weight *c* in *W * by a set of genomes {*g*_1_,⋯*g*_*c*_}, the *expanded gene treeW*_*exp*_obtained from *W * by replacing each leaf *g* with weight *c*>1 by an *expanded leaf *, *i.e.* a caterpillar tree of size *c* containing the leaves *g*_1_,⋯*g*_*c*_(i.e. the tree (*g*_*c*_,(⋯*g*_3_,(*g*_2_,*g*_1_)⋯ )), and the *expanded species treeS*_*exp*_obtained from *S* by replacing each leaf *g* with weight *c*>1 in *W * by a caterpillar tree of size *c* containing the leaves *g*_1_,⋯*g*_*c*_. Then *W*_*exp*_and *S*_*exp*_are uniquely leaf-labelled trees. It is easy to see that a solution $W_{\exp}^{\mathrm{MAX}}$ of MAST will contain, for any *g* ∈ *G*, either *c* or 0 leaves labelled *g* (*i.e.* either 0 or all leaves labelled *g* removed from *W*_*exp*_). Therefore the *compressed* tree $W_{\exp}^{\mathrm{MAX}}$, obtained by recovering a single weighted leaf from each *expanded leaf * of *W*_*exp*_that was not removed by MAST, is a solution to WMAST. Further, since we add at most a constant number of genes per leaf, we will affect the running time of MAST by at most a constant factor. □

## Empirical results

We test the optimality of Algorithm Correct-Tree in the case of a gene tree satisfying Property AD-above-NAD (*i.e.* containing at least one AD vertex above a NAD vertex). Indeed, the algorithm is guaranteed to give the optimal solution otherwise (*i.e.* for trees satisfying the constraints of Section “Uniquely leaf-labelled gene trees” or Section “No AD above NAD”). We compared the number *N* of leaf-removals obtained by Algorithm Correct-Tree with the number *N*_*opt*_obtained by the exact algorithm that tries all possible leaf-subset removals. More precisely, if the minimum number of leaf-removals output by Algorithm Correct-Tree is *r*, we try all subsets of *r*−1,*r*−2,…,*r*−*i* leaf removals, and stop as soon as a tree that is MD-consistent with *S* is obtained. As the naive algorithm has clearly an exponential-time complexity, tests are performed on trees of limited size.

**Figure 4:**
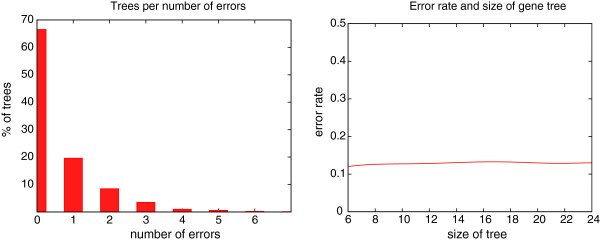
**Comparison of the number *N *of leaf-removal obtained by Algorithm Correct-Tree with the optimal number *N***_***opt ***_**obtained by an exact algorithm.** Left: Percentage of trees leading to a given number *N*−*N*_*opt*_of errors (see text for more details on the used parameters). Right: The error rate, computed as (*N*−*N*_*opt*_)/*N*, depending on the size of the gene tree (number of leaves).

We considered a genome set of fixed size 5, and gene trees with 6 to 24 leaves. For each size *s* (from 6 to 24, with steps of 2), we generated 500 random gene trees of *s* leaves, and kept only those satisfying Property AD-above-NAD. The left diagram of Figure 4 shows that Algorithm Correct-Tree gives an exact solution for more than 65% of the trees (among all of those satisfying Property AD-above-NAD). Moreover, when *N*_*opt*_ differs from *N*_*opt*_, in most cases the difference is 1. The right diagram of Figure 4 is obtained by averaging, for each size *s*, the results obtained for all the gene trees of that size. We can see that the error-rate, computed as (*N*−*N*_*opt*_/*N*, is independent from the size of the tree, and did not exceed 0.15, based on our simulation parameters. After testing other dependency factors (non-shown results), it appears that the error-rate only depends on the number of times the loop 9–13 of Algorithm Correct-Tree is executed, which is not directly related to the number of NADs or ADs in the tree.

**Figure 5:**
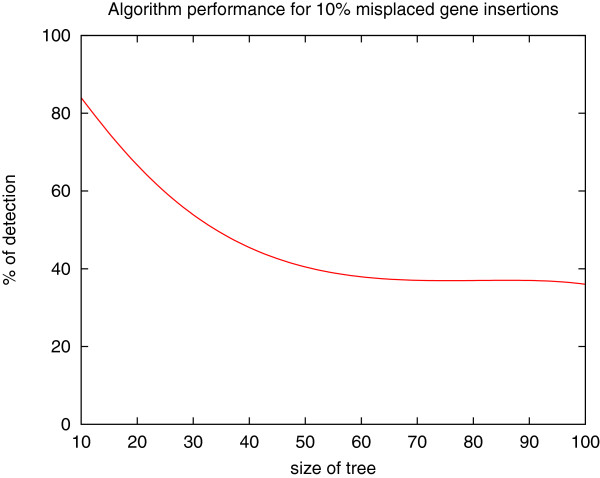
**Percentage of misplaced leaf detection, computed as *(N/NbAdded)×100*, where *NbAdded *is the number of randomly added leaves, and *N *is the number of leaf removals obtained by ALGORITHM CORRECT-TREE (see text for more details).**

Finally, we tested the ability of the approach to identify misplaced genes. To do so, we considered a genome set of fixed size 10, and gene trees of size *s* varying from 10 to 100 (with a step of 10). For a random species tree *S* and a random tree *T* of size *s* that is MD-consistent with *S*, we incorporate randomly *NbAdded*=*s*/10 leaves with randomly chosen labels. We then test how many “misplaced” leaves our method is able to detect. For each size *s*, results are averaged over 100 trees. Figure 5 shows the detection percentage of Algorithm Correct-Tree, which is computed as (*N*/*NbAdded*)×100. This detection percentage decreases with increasing size of the gene tree. This is mainly due to the fact that as an MD-consistent tree needs no leaf removal, its detection percentage is always 100%, and that the more leaves we add (1 for a gene tree of size 10, but 10 for a gene tree of size 100) the less chance we have to end up with an MD-consistent tree. Removing the cases of MD-consistent trees lead to a detection percentage around 40%.

## Conclusion

Based on observations pointing to NAD vertices of a gene tree as indicating potentially misplaced genes, we developed a polynomial-time algorithm for inferring the minimum number of leaf-removals required to transform a gene tree into an MD-tree, *i.e.* a tree with no NAD vertices. The algorithm is exact in the case of a uniquely leaf-labelled gene tree, or in the case of a gene tree that does not contain any AD vertex above a NAD vertex. In the general case, our algorithm exhibited near-optimal results under our simulation parameters. Unfortunately, NAD vertices can only reveal a subset of misplaced genes, as a randomly placed gene does not necessarily lead to a NAD vertex. Our experiments show that, on average, we are able to infer 40% of misplaced genes. However, the additional damage caused by a misplaced leaf leading to a NAD is an excessive increase of the real mutation-cost of the tree. Therefore, removing NADs can be seen as a preprocessing of the gene tree preceding a reconciliation approach, in order to obtain a better view of the duplication-loss history of the gene family.

Another use of our method would be to choose, among a set of equally supported gene trees output by a given phylogenetic method, the one that can be transformed to an MD-consistent tree by a minimum number of leaf removals.

A limitation of our approach is that a NAD resulting from a wrong bipartition {*a*,*b*;*c*} can be, *a priori*, solved by removing any gene from this bipartition. Our present approach is able to detect a number of misplaced genes but, in general, it is insufficient to detect precisely the genes that have been erroneously added in the tree. An extension would be to infer all optimal subsets of leaf removals, and to use bootstrapping values on the edges of the tree for a judicious choice of the genes to be removed.

## Competing interests

The authors declare that they have no competing interests.

## Authors’ contributions

All authors read and approved the final manuscript.

## Funding

Research supported by grants to N.E.M. from the Natural Sciences and Engineering Research Council of Canada, and “Fonds de Recherche Nature et Technologie” of Quebec.
